# U.S. Children “Learning Online” during COVID-19 without the Internet or a Computer: Visualizing the Gradient by Race/Ethnicity and Parental Educational Attainment

**DOI:** 10.1177/2378023121992607

**Published:** 2021-02-17

**Authors:** Joseph Friedman, Hunter York, Ali H. Mokdad, Emmanuela Gakidou

**Affiliations:** 1 University of California, Los Angeles, Los Angeles, CA, USA; 2 Princeton University, Princeton, NJ, USA; 3 University of Washington, Seattle, WA, USA

**Keywords:** educational inequality, online learning, COVID-19, racial/ethnic disparities

## Abstract

The coronavirus disease 2019 pandemic has caused unprecedented disruptions to education in the United States, with a large proportion of schooling moving to online formats, which has the potential to exacerbate existing racial/ethnic and socioeconomic disparities in learning. The authors visualize access to online learning technologies using data from the Household Pulse Survey from the early fall 2020 school period (August 19 to October 26). The authors find that 10.1 percent of children participating in online learning nationally did not have adequate access to the Internet and a computer. Rates of inadequate access varied nearly 20-fold across the gradient of parental race/ethnicity and education, from 1.9 percent for children of Asian parents with graduate degrees to 35.5 percent among children of Black parents with less than a high school education. These findings indicate alarming gaps in potential learning among U.S. children. Renewed investments in equitable access to distance-learning resources will be necessary to prevent widening racial/ethnic and class learning disparities.

The coronavirus disease 2019 (COVID-19) pandemic has caused unprecedented disruptions to education globally and in the United States, with a large proportion of schooling moving to distance-learning formats that require access to the Internet and computer technology. We use data from the weekly Household Pulse Survey (U.S. Census Bureau 2020), a rapidly deployed, representative sample of the U.S. population, describing early trends in the fall 2020 school period (August 19 to October 26). We estimate that 58.1 percent (95 percent confidence interval [CI] = 57.5 percent to 58.7 percent) of U.S. children participated in online learning. Of these, 10.1 percent (95 percent CI = 9.6 percent to 10.6 percent) did not have adequate access to both the Internet and a computer or other electronic device used for educational purposes (Figure 1; see supplement for sample sizes and confidence intervals).

**Figure 1.**
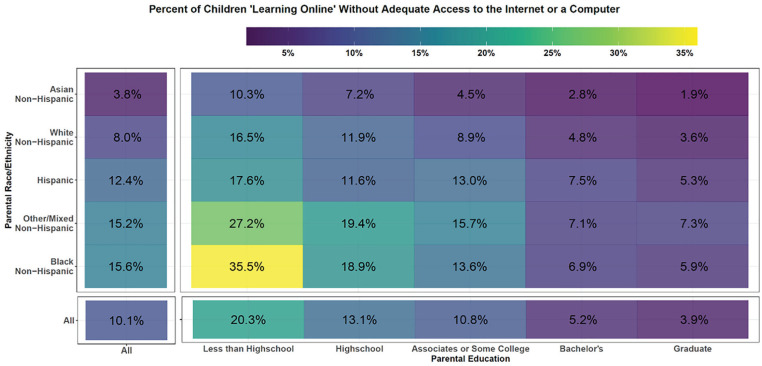
Percentage of children (ages 0–18 years) learning online whose parents reported that they had inadequate access to the Internet or a computer or other electronic device used for educational purposes in the fall 2020 school period (August 19 to October 26), shown by parental race and parental education gradient. All intersections are shown as well as marginal values. All percentages represent survey-weighted values. See the supplement for confidence intervals and more details regarding methodology.

## The Gradient by Parental Race/Ethnicity and Education

Lack of adequate access varied nearly 20-fold across the gradient of parental race/ethnicity and education, ranging from 1.9 percent (95 percent CI = 1.2 percent to 2.7 percent) for children of Asian parents with graduate degrees to 35.5 percent (95 percent CI = 24.1 percent to 47.0 percent) among children of Black parents with less than a high school education (Figure 1).

These rates varied fourfold by race/ethnicity, ranging from 3.8 percent (95 percent CI = 2.8 percent to 4.9 percent) for Asian parents to 15.6 percent (95 percent CI = 13.5 percent to 17.7 percent) for Black parents, and fivefold by parental education, ranging from 3.9 percent (95 percent CI = 3.5 percent to 4.3 percent) for parents with graduate degrees to 20.3 percent (95 percent CI = 16.5 percent to 24.2 percent) for parents with less than high school education.

## Implications

Even before COVID-19, the United States had profound and persistent disparities in educational attainment and learning by race and social class (York 2020). The results presented here concur with those of recent studies suggesting that COVID-19 may be further exacerbating existing gaps in schooling (Bacher-Hicks, Goodman, and Mulhern 2020) and extends them to the fall 2020 school period. Children expected to participate in online learning without adequate technology are highly unlikely to achieve significant learning compared with their peers to whom more resources are available. Falling behind in learning goals, they may also be more likely to leave school entirely.

By visualizing the gradient by parental race/ethnicity and education, we highlight that COVID-19 is likely potentiating the intergenerational propagation of gaps in educational outcomes and moreover in a differential manner by race/ethnicity. These findings therefore demonstrate that disparities in remote learning during COVID-19 must be examined in an intersectional fashion (Bhopal and Preston 2012). Stratification by parental race and education revealed disparities along both dimensions, with specific subgroups exposed to large magnitude disparities. Future studies should examine how racial/ethnic and social class differences also may vary by geography, potentially reflecting the diversity of educational approaches taken by municipal and state government in response to COVID-19-related disruptions.

As education is a key social determinant of health, and a driver of economic opportunities, the implications of these widening education gaps are myriad and may ripple out into disparities in numerous sectors of society (Lim et al. 2018). These trends highlight a need for renewed investments in ensuring universal access to distance-learning resources for all children in the United States and may have implications for school districts and states making decisions regarding school closures.

## Supplemental Material

sj-docx-1-srd-10.1177_2378023121992607 – Supplemental material for U.S. Children “Learning Online” during COVID-19 without the Internet or a Computer: Visualizing the Gradient by Race/Ethnicity and Parental Educational Attainment Click here for additional data file. Supplemental material, sj-docx-1-srd-10.1177_2378023121992607 for U.S. Children “Learning Online” during COVID-19 without the Internet or a Computer: Visualizing the Gradient by Race/Ethnicity and Parental Educational Attainment by Joseph Friedman, Hunter York, Ali H. Mokdad and Emmanuela Gakidou in Socius
