# Supplementary material for: U.S. Children “Learning Online” during COVID-19 without the Internet or a Computer: Visualizing the Gradient by Race/Ethnicity and Parental Educational Attainment
Source: Socius. 2021 Feb 17;7:2378023121992607. doi: 10.1177/2378023121992607 (PMC7890417; doi:10.1177/2378023121992607)
Supplement: sj-docx-1-srd-10.1177_2378023121992607 – Supplemental material for U.S. Children “Learning Online” during COVID-19 without the Internet or a Computer: Visualizing the Gradient by Race/Ethnicity and Parental Educational Attainment [file sj-docx-1-srd-10.1177_2378023121992607.docx]

**Supplemental Materials for: “US Children ‘Learning Online’ During COVID-19 Without the Internet or a Computer: Visualizing the Gradient by Race and Parental Educational”**

**Steps Taken to Conduct the Analysis**

Public use microdata from the weekly Household Pulse Survey were obtained from the US Census Bureau: [*https://www.census.gov/programs-surveys/household-pulse-survey/datasets.html*](https://www.census.gov/programs-surveys/household-pulse-survey/datasets.html) *(accessed Nov 25, 2020).*

1. Data from waves 13-17 were used, as they ask explicitly about the 2020-2021 school year.
2. Parental race and ethnicity were categorized into 5 groups using the *RHISPANIC* and *RRACE* variables. Race and ethnicity variables had no observed missingness.
3. Parental income was excluded as an analytical variable due to high missingness (22%).
4. Parental education was included as an analytical variable given no apparent missingness, and high relevance to the study topic. It was categorized into 5 categories from the variable *EEDUC*.
5. A subset of the full dataset was used, consisting of only those households with at least 1 child currently enrolled in school, who answered affirmatively to the *TEACH2* question: *“Classes normally taught in person moved to a distance-learning format using online resources, either self-paced or in real time.”*
6. The outcome variable was defined using the variables *COMPAVAIL* and *INTRNTAVAIL* describing children’s computer/other electronic device and internet access, respectively. Households reporting that both were “always available” or “usually available” were assigned a value of 0, whereas for households in which either variable was reported as “sometimes available” “rarely available” and “never available” a value of 1 was assigned.
7. Missingness in either variable (*COMPAVAIL* and *INTRNTAVAIL*) was low, at 0.019% of the subset of households with children participating in online learning.
8. Percentages were calculated for all values shown in the figure. Survey weights were used for all values. The weights were calculated by using each household weight, multiplied by the number of children in the house, to count all children equally.
9. Standard errors for percentages were calculated according the methodology specified by the US Census Bureau: *https://www2.census.gov/programs-surveys/demo/technical-documentation/hhp/Phase2_Source_and_Accuracy_Week_17.pdf (accessed November 25, 2020).* This entailed using a weights file produced by the Census Bureau, containing 80 household weights for each respondent. Each outcome for each group was initially calculated using the main household weight to create the point estimate. Subsequently, each outcome was calculated 80 times, using each of the 80 household weights. The standard errors for each estimate was generated using the below formula, where $\hat{\theta}$ is the point estimate and $\theta_{i}$is the estimate calculated from each alternative household weight:

$$Standard Error \left( \hat{\theta} \right)=sqrt( \frac{4}{80} \sum_{i=1}^{80} \left( \theta_{i}- \hat{\theta} \right)^{2})$$

**Methodological Considerations**

1. Percentages reflect US households surveyed in the August 19 – October 26 window, from waves 13 through 17 of the survey. Each wave of the survey is equally weighted. The total sample size for this period was n= 502,692 respondents. The sample size of the subset of households with children actively participating in online learning was n= 72,179 respondents. See all sample sizes for each subgroup in the supplemental table below.
2. Standard errors were created by the Census Bureau using successive difference replication. They primary capture the magnitude of sampling error, although some non-sampling error is additionally measured. Bias introduced by non-sampling error is not captured.
3. All questions were answered by one representative from each household. In households with multiple children, there could be nuances between children that are missed by the survey design. It is likely reasonable to assume that most children in the household have similar access to the internet and computer technology. It may also be reasonable to assume that online-learning decisions affect children in the household equally. However, this does represent a limitation that should be taken into consideration when interpreting study results.
4. The ‘COMPAVAIL’ question has the description “Computer availability for educational purposes” and is worded “How often is a computer or other digital device available to children for educational purposes?”. This wording has some vagueness in the nature of the digital device being used for educational purposes. Some respondents may consider certain devices adequate, in instances where an expert may not agree. However, it is not possible to disaggregate by type of device using the current dataset.
5. More survey methodological details are available here: [*https://www.census.gov/programs-surveys/household-pulse-survey/technical-documentation.html*](https://www.census.gov/programs-surveys/household-pulse-survey/technical-documentation.html) *(accessed November 25, 2020).*
6. Additionally, it is important to note that further research will be required to more directly measure learning gaps stemming from COVID-19 related educational disruptions. While important, access to the internet and a computer are only a proxy for learning outcomes.

| Race/Ethnicity Group | Parental Education | Sample Size (# of Children) | Estimate (95% CI) |
| --- | --- | --- | --- |
| All | All | 72179 (140367) | 10.1% ( 9.6% - 10.6%) |
| All | Graduate | 20254 (39052) | 3.9% ( 3.5% - 4.3%) |
| All | Bachelor's | 21713 (41940) | 5.2% ( 4.6% - 5.9%) |
| All | Associates or Some College | 21853 (42450) | 10.8% (10.0% - 11.6%) |
| All | Highschool | 6940 (13774) | 13.1% (12.0% - 14.3%) |
| All | Less than Highschool | 1419 (3151) | 20.3% (16.5% - 24.2%) |
| Asian, Non-Hispanic | All | 5049 (9131) | 3.8% ( 2.8% - 4.9%) |
| Asian, Non-Hispanic | Graduate | 2123 (3769) | 1.9% ( 1.2% - 2.7%) |
| Asian, Non-Hispanic | Bachelor's | 1803 (3257) | 2.8% ( 2.0% - 3.5%) |
| Asian, Non-Hispanic | Associates or Some College | 822 (1526) | 4.5% ( 2.5% - 6.6%) |
| Asian, Non-Hispanic | Highschool | 225 (427) | 7.2% ( 1.8% - 12.7%) |
| Asian, Non-Hispanic | Less than Highschool | 76 (152) | 10.3% ( 3.5% - 17.0%) |
| White, Non-Hispanic | All | 48421 (94215) | 8.0% ( 7.5% - 8.5%) |
| White, Non-Hispanic | Graduate | 14461 (28336) | 3.6% ( 3.1% - 4.1%) |
| White, Non-Hispanic | Bachelor's | 15682 (30758) | 4.8% ( 4.1% - 5.5%) |
| White, Non-Hispanic | Associates or Some College | 13694 (26236) | 8.9% ( 8.1% - 9.6%) |
| White, Non-Hispanic | Highschool | 4067 (7799) | 11.9% (10.5% - 13.3%) |
| White, Non-Hispanic | Less than Highschool | 517 (1086) | 16.5% (10.8% - 22.2%) |
| Hispanic | All | 8910 (17873) | 12.4% (10.8% - 13.9%) |
| Hispanic | Graduate | 1461 (2840) | 5.3% ( 3.7% - 6.9%) |
| Hispanic | Bachelor's | 1970 (3703) | 7.5% ( 4.7% - 10.3%) |
| Hispanic | Associates or Some College | 3439 (6915) | 13.0% (10.9% - 15.2%) |
| Hispanic | Highschool | 1439 (3055) | 11.6% ( 9.2% - 14.0%) |
| Hispanic | Less than Highschool | 601 (1360) | 17.6% (11.5% - 23.7%) |
| Other/Mixed, Non-Hispanic | All | 3310 (6768) | 15.2% (12.5% - 17.9%) |
| Other/Mixed, Non-Hispanic | Graduate | 653 (1285) | 7.3% ( 4.0% - 10.7%) |
| Other/Mixed, Non-Hispanic | Bachelor's | 835 (1646) | 7.1% ( 4.0% - 10.3%) |
| Other/Mixed, Non-Hispanic | Associates or Some College | 1324 (2731) | 15.7% (11.9% - 19.5%) |
| Other/Mixed, Non-Hispanic | Highschool | 431 (934) | 19.4% (13.0% - 25.9%) |
| Other/Mixed, Non-Hispanic | Less than Highschool | 67 (172) | 27.2% ( 7.8% - 46.6%) |
| Black, Non-Hispanic | All | 6489 (12380) | 15.6% (13.5% - 17.7%) |
| Black, Non-Hispanic | Graduate | 1556 (2822) | 5.9% ( 4.1% - 7.8%) |
| Black, Non-Hispanic | Bachelor's | 1423 (2576) | 6.9% ( 4.6% - 9.3%) |
| Black, Non-Hispanic | Associates or Some College | 2574 (5042) | 13.6% (11.1% - 16.2%) |
| Black, Non-Hispanic | Highschool | 778 (1559) | 18.9% (14.6% - 23.1%) |
| Black, Non-Hispanic | Less than Highschool | 158 (381) | 35.5% (24.1% - 47.0%) |

**Supplemental Table.** Percent of children (age 0-18) learning online whose parents reported they had inadequate access to the internet or a computer, in the Fall 2020 school period (August 31 – October 26), shown by parental race and parental education gradient. All intersections are shown as well as marginal values. All percentages represent survey-weighted values. The sample size is shown, representing the number of respondents, alongside the number of children living with those respondents, shown in parentheses. The point estimate is shown along a 95% confidence interval for each quantity.

**Data and Code Access**

All data used in the analysis are publicly available at: [*https://www.census.gov/programs-surveys/household-pulse-survey/datasets.html*](https://www.census.gov/programs-surveys/household-pulse-survey/datasets.html) *(accessed Nov 25, 2020).*

The R code used to run the analysis is available below (to be made available on a public GitHub page, along with the data, for publication). Note that the root directory must be set the same location as the downloaded data, to use the code.

##-----------------------------------------------------------------##

## Online Learning Parental Race and Education Case Study

## Setup

rm(list = ls())

pacman::p_load(data.table, tidyverse, ggplot2,ggrepel, grid, gridExtra,lubridate,reldist,cowplot)

root <- <<< SET ME>>>>>

#--------------------load data--------------------#

#weeks 13-17 refer to 2020-2021 school year

dat <- rbindlist(lapply(FUN=fread,(paste0(root,"data/HPS_Week",13:17,"_PUF_CSV/pulse2020_puf_",13:17,".csv"))))

#80 weights for each respondent

wts <- rbindlist(lapply(FUN=fread,(paste0(root,"data/HPS_Week",13:17,"_PUF_CSV/pulse2020_repwgt_puf_",13:17,".csv"))))

dat <- merge(dat,wts,by="SCRAM")

#is each week evenly weighted? YES

dat[,.(PWEIGHT=sum(PWEIGHT)),by=.(WEEK)]

#--------------------Prep Variables--------------------#

#---Stratifiers---#

#-race/ethn

table(dat$RHISPANIC,useNA = "always")

table(dat$RRACE,useNA = "always")

dat[RHISPANIC==2,group:="Hispanic"]

dat[RHISPANIC==1 & RRACE==1,group:="White\nNon-Hispanic"]

dat[RHISPANIC==1 & RRACE==2,group:="Black\nNon-Hispanic"]

dat[RHISPANIC==1 & RRACE==3,group:="Asian\nNon-Hispanic"]

dat[RHISPANIC==1 & RRACE==4,group:="Other/Mixed\nNon-Hispanic"]

table(dat$group,useNA = "always")

#-household income

table(dat$INCOME,useNA = "always")

nrow(dat[INCOME<0]) / nrow(dat) #22% missing

#-parental education

table(dat$EEDUC,useNA = "always") #no apparrent missingness

dat[EEDUC<3,PEDU:="Less than Highschool"]

dat[EEDUC%in%c(3),PEDU:="Highschool"]

dat[EEDUC%in%c(4,5),PEDU:="Associates or Some College"]

dat[EEDUC%in%c(6),PEDU:="Bachelor's"]

dat[EEDUC%in%c(7),PEDU:="Graduate"]

"1) Less than high school

2) Some high school

3) High school graduate or equivalent (for example GED)

4) Some college, but degree not received or is in progress

5) Associate's degree (for example AA, AS)

6) Bachelor's degree (for example BA, BS, AB)

7) Graduate degree (for example master's, professional, doctorate)"

#number of kids

table(dat$THHLD_NUMKID,useNA = "always") #no apparrent missingness

dat[,numk:=THHLD_NUMKID]

#---Outcome---#

#no computer or internet, among only those distance learning

dat[TEACH2==1 & (COMPAVAIL %in% c(1,2) & INTRNTAVAIL %in% c(1,2)),dist_noresource_prop:=0]

dat[TEACH2==1 & (COMPAVAIL %in% c(3,4,5) | INTRNTAVAIL %in% c(3,4,5)),dist_noresource_prop:=1]

#%online learning

dat[,dist:=0]

dat[TEACH2==1,dist:=1]

#percent of kids in online school

# online <- dat[numk>0&(ENROLL1==1|ENROLL2==1)]

# weighted.mean(x=online$dist,w=online$HWEIGHT*online$numk)

#subset to only parents with >0 children, who report their children are distance-learning

kid <- dat[(numk>0&(ENROLL1==1|ENROLL2==1))]

#Check Missingness

nrow(kid[is.na(dist_noresource_prop)]) / nrow(kid) * 100

#subset to only needed variables

kid <- kid[,c("dist_noresource_prop","dist","numk","group","PEDU","HWEIGHT",paste0("HWEIGHT",1:80))]

#long on weights

kid <- melt.data.table(kid,id.vars=c("dist_noresource_prop","dist","numk","group","PEDU"),variable.name = "weight_n",value.name = "weight")

#--------------------Collapse--------------------#

kid[,obs:=1]

#intersection

kc.re <- kid[,.(ss=sum(obs),numk=sum(numk),

dist_noresource_prop=weighted.mean(x=dist_noresource_prop,w=weight*numk,na.rm=T),

dist=weighted.mean(x=dist,w=weight*numk,na.rm=T)

),by=.(group,PEDU,weight_n)]

#parental education

kc.e <- kid[,.(ss=sum(obs),numk=sum(numk),

dist_noresource_prop=weighted.mean(x=dist_noresource_prop,w=weight*numk,na.rm=T),

dist=weighted.mean(x=dist,w=weight*numk,na.rm=T)

),by=.(PEDU,weight_n)]

kc.e[,group:="All"]

#parental race

kc.r <- kid[,.(ss=sum(obs),numk=sum(numk),

dist_noresource_prop=weighted.mean(x=dist_noresource_prop,w=weight*numk,na.rm=T),

dist=weighted.mean(x=dist,w=weight*numk,na.rm=T)

),by=.(group,weight_n)]

kc.r[,PEDU:="All"]

#total

kc <- kid[,.(ss=sum(obs),numk=sum(numk),

dist_noresource_prop=weighted.mean(x=dist_noresource_prop,w=weight*numk,na.rm=T),

dist=weighted.mean(x=dist,w=weight*numk,na.rm=T))

,by=.(weight_n)]

kc[,PEDU:="All"]

kc[,group:="All"]

#append all aggs

aggs <- rbind(kc.re,kc.r,kc.e,kc)

#long on variable

aggs.l <- melt.data.table(aggs,id.vars=c("group","PEDU","weight_n","ss","numk"))

#get point estimate wide

aggs.l[weight_n=="HWEIGHT",value_mean:=value]

aggs.l[,value_mean:=mean(value_mean,na.rm=T),by=.(group,PEDU,variable)]

aggs.l <- aggs.l[weight_n!="HWEIGHT"]

#get squared differences

aggs.l[,dif_sq:=(value-value_mean)^2]

#sum them

aggs.l <- aggs.l[,.(sum_dif_sq=sum(dif_sq)),by=.(group,PEDU,ss,numk,value_mean,variable)]

#multiply by 4/80

aggs.l[,value_var:=(4/80)*sum_dif_sq]

#take square root to get standard error

aggs.l[,value_se:=sqrt(value_var)]

#create 95%CI

aggs.l[,value_upr:=value_mean+(1.96*value_se)]

aggs.l[,value_lwr:=value_mean-(1.96*value_se)]

#Define stratifier order

aggs.l[,PEDU:=factor(PEDU,levels=c("Less than Highschool","Highschool","Associates or Some College","Bachelor's","Graduate","All"))]

aggs.l[,group:=factor(group,levels=c( "Black\nNon-Hispanic","Other/Mixed\nNon-Hispanic","Hispanic","White\nNon-Hispanic","Asian\nNon-Hispanic","All"))]

#--------------------Graph Disparities--------------------#

#c.var <- "dist"

#c.var <- "dist_noresource_prop"

var.name <- data.table(variable=c("dist_noresource_prop","dist"),

varname=c("Percent of Children 'Learning Online' Without Adequate Access to the Internet or a Computer",

"Percent of Children Participating in Online Learning"))

pdf(paste0(root,"/visuals/Online_Learning_Race_Edu_Gradient.pdf"),width=17,height=8)

for (c.var in unique(aggs.l$variable)) {

c.min <- min(aggs.l[variable==c.var,value_mean*100])*.99

c.max <- max(aggs.l[variable==c.var,value_mean*100])*1.01

gg1a <- ggplot(aggs.l[variable==c.var&group!="All"&PEDU!="All"],

aes(y=group,x=PEDU,fill=value_mean*100,label=paste0(format(round(value_mean*100,1),nsmall=1),"%"))) +

geom_tile(alpha=.8) + geom_text(size=7) +

theme_bw() +

scale_fill_viridis_c(name="",breaks=seq(0,40,5),labels=paste0(seq(0,40,5),"%"),limits=c(c.min,c.max),guide=guide_colorbar(barwidth=50,barheight=2)) +

theme(

legend.text = element_text(size=14,face="bold"),

legend.title = element_text(size=14,face="bold"),

strip.background = element_rect(fill="white"),

legend.position = "top",

axis.text.x = element_blank(),axis.ticks.x=element_blank(),axis.title.x = element_blank(),

axis.text.y = element_blank(),axis.ticks.y=element_blank(),axis.title.y = element_blank(),

plot.margin=unit(c(.5,.5,0,0), "cm")) +

labs(y="",x="")

#Race Margins

gg1b <- ggplot(aggs.l[variable==c.var&group!="All"&PEDU=="All"],

aes(y=group,x=PEDU,fill=value_mean*100,label=paste0(format(round(value_mean*100,1),nsmall=1),"%"))) +

geom_tile(alpha=.8) + geom_text(size=7) +

theme_bw() +

scale_fill_viridis_c(name="",limits=c(c.min,c.max),guide=guide_colorbar(barwidth=30,barheight=1)) +

theme(

strip.background = element_rect(fill="white"),

legend.position = "none",

axis.text.y=element_text(face="bold",size=14,angle=0),axis.title.y=element_text(face="bold",size=14),

axis.text.x = element_blank(),axis.ticks.x=element_blank()) +

labs(y="Parental Race/Ethnicity",x="")

#Education Margins

gg1c <- ggplot(aggs.l[variable==c.var&group=="All"&PEDU!="All"],

aes(y=group,x=PEDU,fill=value_mean*100,label=paste0(format(round(value_mean*100,1),nsmall=1),"%"))) +

geom_tile(alpha=.8) + geom_text(size=7) +

theme_bw() +

scale_fill_viridis_c(name="",limits=c(c.min,c.max),guide=guide_colorbar(barwidth=30,barheight=1)) +

theme(

strip.background = element_rect(fill="white"),

legend.position = "none",

axis.text.x=element_text(face="bold",size=14),axis.title.x=element_text(face="bold",size=14),

axis.text.y = element_blank(),axis.ticks.x=element_blank(),

plot.margin=unit(c(-0.5,.5,0,0), "cm")) +

labs(x="Parental Education",y="")

#Total Margin

gg1d <- ggplot(aggs.l[variable==c.var&group=="All"&PEDU=="All"], aes(y=" All",x="All",fill=value_mean*100,label=paste0(format(round(value_mean*100,1),nsmall=1),"%"))) +

geom_tile(alpha=.8) + geom_text(size=7) +

theme_bw() +

scale_fill_viridis_c(name="",limits=c(c.min,c.max),guide=guide_colorbar(barwidth=30,barheight=1)) +

theme(

strip.background = element_rect(fill="white"),

legend.position = "none",

axis.text.x=element_text(face="bold",size=14),

axis.text.y = element_text(face="bold",size=14,angle=0),

plot.margin=unit(c(-0.5,-.2,0,0), "cm")) +

labs(x="",y="")

p1 <- plot_grid(gg1b,gg1a,align="h",axis="tb",rel_widths = c(.3,1))

lay = rbind(c(1,1,1,1,1,1,1,1,1),

c(1,1,1,1,1,1,1,1,1),

c(1,1,1,1,1,1,1,1,1),

c(1,1,1,1,1,1,1,1,1),

c(1,1,1,1,1,1,1,1,1),

c(3,3,2,2,2,2,2,2,2))

c.title <- var.name[variable==c.var,varname]

p2 <- grid.arrange(p1,gg1c,gg1d,layout_matrix=lay,

top=textGrob(c.title,

gp=gpar(fontsize=17,fontface="bold"),hjust=.5))

}

dev.off()

#Supplemental Table - Sample Size in Each Group

aggs.l[,sample:=paste0(ss," (",numk,")")]

aggs.l[,estimate:=paste0(format(round(value_mean*100,1),nsmall=1),"% (",

format(round(value_lwr*100,1),nsmall=1),"% - ",

format(round(value_upr*100,1),nsmall=1),"%)")]

tbl <- aggs.l[,c("group","PEDU","variable","sample","estimate")]

tbl <- tbl[order(-group,-PEDU)]

tbl[,group:=gsub(group,pattern="\n",replacement=", ")]

View(tbl)

write.csv(tbl,paste0(root,"visuals/supplemental_table.csv"),row.names=F)
